# Continuity of mental health care during the transition from prison to the community following brief periods of imprisonment

**DOI:** 10.3389/fpsyt.2022.934837

**Published:** 2022-09-20

**Authors:** Christie C. Browne, Daria Korobanova, Prabin Chemjong, Anthony W. F. Harris, Nick Glozier, John Basson, Sarah-Jane Spencer, Kimberlie Dean

**Affiliations:** ^1^Faculty of Medicine and Health, University of New South Wales, Sydney, NSW, Australia; ^2^Justice Health and Forensic Mental Health Network, Sydney, NSW, Australia; ^3^Western Sydney Local Health District, Sydney, NSW, Australia; ^4^Faculty of Medicine and Health, University of Sydney, Sydney, NSW, Australia; ^5^Sydney Local Health District, Sydney, NSW, Australia; ^6^Australian Research Council Centre of Excellence for Children and Families Over the Life Course, Indooroopilly, QLD, Australia

**Keywords:** prison, transition, mental health, community, release, reoffending

## Abstract

**Purpose:**

The prison-to-community transition period is one of high risk and need, particularly for those with mental illness. Some individuals cycle in and out of prison for short periods with little opportunity for mental health stabilization or service planning either in prison or the community. This study describes the socio-demographic, clinical and criminal justice characteristics of individuals with mental illness and frequent, brief periods of imprisonment, examines continuity of mental health care between prison and the community for this group, and reports on their post-release mental health and criminal justice outcomes.

**Design/methodology/approach:**

This study examined a sample of 275 men who had recently entered prison in New South Wales (NSW), Australia, who had been charged with relatively minor offenses and had been identified on reception screening as having significant mental health needs. Baseline demographic and mental health information was collected *via* interview and file review and contacts with the prison mental health service were recorded for the period of incarceration. Follow-up interviews were conducted 3 months post-release to determine level of health service contact and mental health symptoms. Information on criminal justice contact during the 3 month period was also collected.

**Findings:**

The majority (85.5%) of the sample had contact with a mental health professional during their period of incarceration. Mental health discharge planning was, however, lacking, with only one in 20 receiving a referral to a community mental health team (CMHT) and one in eight being referred for any kind of mental health follow-up on release. Of those followed up 3 months post-release (*n* = 113), 14.2% had had contact with a CMHT. Of those released for at least 3 months (*n* = 255), one in three had received new charges in this period and one in five had been reincarcerated.

**Conclusion:**

Continuity of mental health care for those exiting prison is poor, particularly for those with mental health needs experiencing brief periods of imprisonment, and rates of CMHT contact are low in the immediate post-release period. These findings suggest a need for early identification of individuals in this group for timely commencement of intervention and release planning, and opportunities for diversion from prison should be utilized where possible.

## Introduction

Studies of the prevalence of mental illness in prisons worldwide have found higher rates in prison than in the general community (1). In Australia, it is estimated that 40% of individuals in prison have a mental health condition (2), approximately double the rate of 20.1% in the general population (3), and even higher when the relative presence of serious mental illnesses such as psychosis are considered (4). Some individuals with serious mental illness will experience a “revolving door” of multiple short periods of incarceration; one study in the United States finding that those with any major mental disorder diagnosis were nearly two and a half times more likely to experience four or more incarcerations in a 6-year period than those without any diagnosis (5). Amongst those with mental illness in prison, a serious mental illness diagnosis (schizophrenia spectrum/bipolar affective disorder) has been shown to be associated with a higher rate of return to prison within a year of release (6). Short periods of incarceration present little opportunity for mental health stabilization or care planning either in prison or in the community, leading to particularly poor outcomes for individuals and a burden on health and justice systems (5, 6).

Mental health services in prison are consistently assessed as being under-resourced for the level of need (7), and those with mental illness frequently receive inadequate and fragmented care and support both during their time in prison and in the transition period from prison to community. Short sentences are likely to be an important factor; in 2019–2020 the median custodial sentence length in Australia was 9 months, and over a third (35%) of sentences were <6 months long (8). This means that in many cases there is little time to identify and address the factors – including mental ill health – that have contributed to the circumstances leading to imprisonment, and to work collaboratively with public mental health services (in Australia, community mental health teams) to ensure the smooth transition of mental health care from prison to community. Rehabilitation and returning healthier individuals to their communities should be a goal of correctional systems; instead, there is evidence for significant harmful effects of incarceration, which first disrupts and then becomes an ongoing barrier to stable housing, income, support systems and mental health care (9), and in itself increases the risk of re-incarceration for those with mental illness (10).

Despite the critical importance of continuity of care for those with serious mental illness, discharge planning for those transitioning from prison to the community is often inadequate, particularly for unsentenced prisoners where there is uncertainty around discharge (11). Evidence suggests that intervention during this period can improve post-release contact with mental health services in the community (12), which can impact on risk of reoffending. Those who receive outpatient or case management mental health services in the early post release period have been found to be less likely to be reincarcerated within 3 months of release (10) and conversely, a recent study (13) found that treatment disengagement resulted in a threefold increase in risk of reoffending for offenders with psychosis. The relationship between mental health treatment and reoffending, however, is not straightforward, with some studies finding mental health treatment post-release from prison to be associated with an increase in the likelihood of reincarceration, likely due in part to higher levels of monitoring and the confounding effects of illness severity (14, 15).

Whatever the cause, those with serious mental health issues and multiple short-term incarcerations account for a disproportionate amount of overall health service use (6). However, little is known about this group in terms of sociodemographic profile and patterns of mental health care both in prison and post-release. Studies examining outcomes for those with mental illness post-release from prison most often rely on data linkage: such studies have shown that rates of community mental health service contact in the post-release period are low (16), and those with mental illness are more likely to experience poor outcomes related to suicide and self-harm (17, 18) as well as higher mortality rates (19, 20). These studies do not, however, provide information on outcomes for the specific group of those who have both mental illness and short, frequent periods of incarceration. A handful of studies have prospectively followed up participants post release from prison and have found mental health impairment, drug and alcohol use and psychological distress persists beyond the period of incarceration (16, 21). However, most prospective studies of this nature recruit sentenced prisoners due to them having predictable release dates; meaning that those on remand and those who end up spending only short periods in prison are unlikely to be captured.

This study aims to prospectively examine continuity of mental health care for a sample of men with mental illness experiencing a short period of imprisonment, by determining rates of referral to community mental health services on release and rates of community mental health service contact post-release. The socidemographic, clinical and criminal justice characteristics of this group are described, as well as patterns of mental health care both in prison and in the early post-release period. Finally, mental health and criminal justice outcomes at 3 months post release are presented.

## Method

### Setting and sample

The study sample was recruited from the Metropolitan Reception and Remand Center (MRRC) at Silverwater Correctional Complex in Sydney, one of the main reception centers for men entering prison in New South Wales (NSW). All individuals in prison undergo a health screening process upon reception which is completed by a primary care nurse. Health information is recorded and referrals generated to specialist prison health services as needed (e.g., mental health service, general practitioner, drug and alcohol service). Between March 2019 and March 2020, all individuals entering prison at the MRRC during this period were identified through the electronic Patient Administration System (PAS) and were deemed eligible if they had been identified as having significant mental health care needs (as indicated by a referral to prison mental health services following reception screening upon entry), as well having only minor or non-indictable charges (i.e., unlikely to result in a long sentence) or a sentence of <6 months. Individuals were ineligible if they had previously been approached by the researchers for participation in this study during a prior period of incarceration. Each working day during the recruitment period, all eligible individuals who had arrived at the MRRC within the previous 48 h were identified, randomized using a random number generator, and approached in that order. Participation was voluntary and informed consent was obtained to conduct an interview, to obtain collateral information *via* file and electronic databases, and to contact the participant post-release. Participants received $10 if they completed an interview, deposited into their prison account.

At 3 months post-release, participants were contacted for follow-up. For those in the community, this involved calling individuals on the contact number provided at initial interview. If unable to reach participants on their provided number, secondary contacts were contacted for the purpose of obtaining new contact details for the participants. Researchers attempted to contact participants for up to a week before they were classified as “lost to follow-up” if contact was unsuccessful. Individuals who were reincarcerated at the 3 month point were interviewed face-to-face if they were located at MRRC, or by phone at the prison where they were located, facilitated by health staff at that center. Participants received a further $10 at follow-up, in the form of a supermarket voucher if interviewed by phone in the community, and by deposit into their prison account if the follow-up interview occurred when they had returned to prison. Follow-up data collection continued until September, 2020.

### Measures

#### Baseline and follow-up interviews

Interviews of approximately an hour long were completed by two research project officers; a forensic psychologist (CB) and a registered mental health nurse (PC). Interviews consisted of questionnaires developed specifically for the study, recording self-reported socio-demographic, clinical, substance use and criminal justice information, as well as the Prison Mental Health Screening (PMHS) tool (Supplementary file 1), a brief mental health reception screening instrument developed for this study by the researchers. The PMHS covers self-reported history of psychiatric diagnoses, history of mental health treatment, history of suicide attempts and self-harm and current suicidal ideation. The presence of key psychiatric symptoms experienced historically and currently (within the last month) were also elicited and encompassed six symptoms of psychosis (hallucinations, paranoia, unusual beliefs, thought broadcasting, thought interference, ideas of reference) and six mood symptoms (low mood, loss of pleasure, mania, reduced functioning, concentration difficulties, sleep disturbance).

A contact sheet was also completed, to record participants' likely contact details upon release for follow-up purposes, as well as details of a secondary contact person and community mental health team (CMHT) they had had contact with, if relevant.

Following the baseline interview, a file review of the participant's prison health record was conducted to collect collateral sociodemographic, criminal justice and mental health information according to a file review template developed for the purpose of this study (Supplementary file 2) Diagnostic information presented in the current analysis reflects information obtained from the health record, and in the absence of a record (*n* = 25), self-report diagnoses were obtained from the baseline interview.

The 3 month follow-up interview took up to 30 min and utilized a template developed by the research team to complement data collected at baseline (Supplementary file 3). The questionnaire sought information about contact with Community Mental Health Teams and any other mental health services received since release, including therapy or counseling (“In the last 3 months, did you receive any therapy where you and your keyworker or other clinician explored your thoughts, feelings, and beliefs about your symptoms and illness and came up with new ways of understanding them and coping?”). Participants were also asked about recent mental health symptoms (in the same format as at baseline interview), current use of prescribed medication for mental health, and substance use since release.

#### Primary outcome: Continuity of mental health care

Continuity of care was firstly measured by determining the proportion of those participants who were released by the end of the data collection period (*n* = 271) who received a referral from the prison mental health service to a Community Mental Health Team at the time of their release. When participants were released, details of any referrals made to community-based services for the purposes of mental health care were obtained from prison health service (i.e., Justice Health and Forensic Mental Health Network) electronic health records.

The proportion of those in the follow-up group (*n* = 113) who had any contact with a Community Mental Health Team in the 3 months post-release (as reported during the follow-up interview) was the second measure of continuity of care.

#### Secondary outcomes

*Mental health care in prison* was determined by examining contacts with prison mental health services as recorded on the electronic Patient Administration System. For each participant, for a period of 6 months post-reception, details of all mental health contacts were recorded.

*Post-release clinical outcomes* were derived from the 3 month follow-up interview and examined mental health symptoms, medication and substance use since release.

*Reoffending outcomes* were determined 3 months post-release through Corrective Services NSW electronic records. New charges were recorded as well as whether the participant was reincarcerated in the 3 month period (defined as returning to prison custody, not including police cells).

### Statistical analysis

Statistical analyses were conducted using SPSS Statistics 27. Descriptive statistics for the overall sample and the follow-up sample were obtained and Chi-Square analyses used to determine differences between those followed up at 3 months and those not followed up with regard to key socio-demographic, criminal justice and mental health variables. A figure was produced to present the proportion of released participants who received a referral for mental health care, and the proportion of those followed up who had contact with a Community Mental Health Team. Chi-square and logistic regression analyses were conducted to make comparisons between the group referred to mental health services when released and those who were not referred, as well as those reincarcerated within 3 months vs. those who were not.

## Results

### Results of recruitment

**Figure 1:**
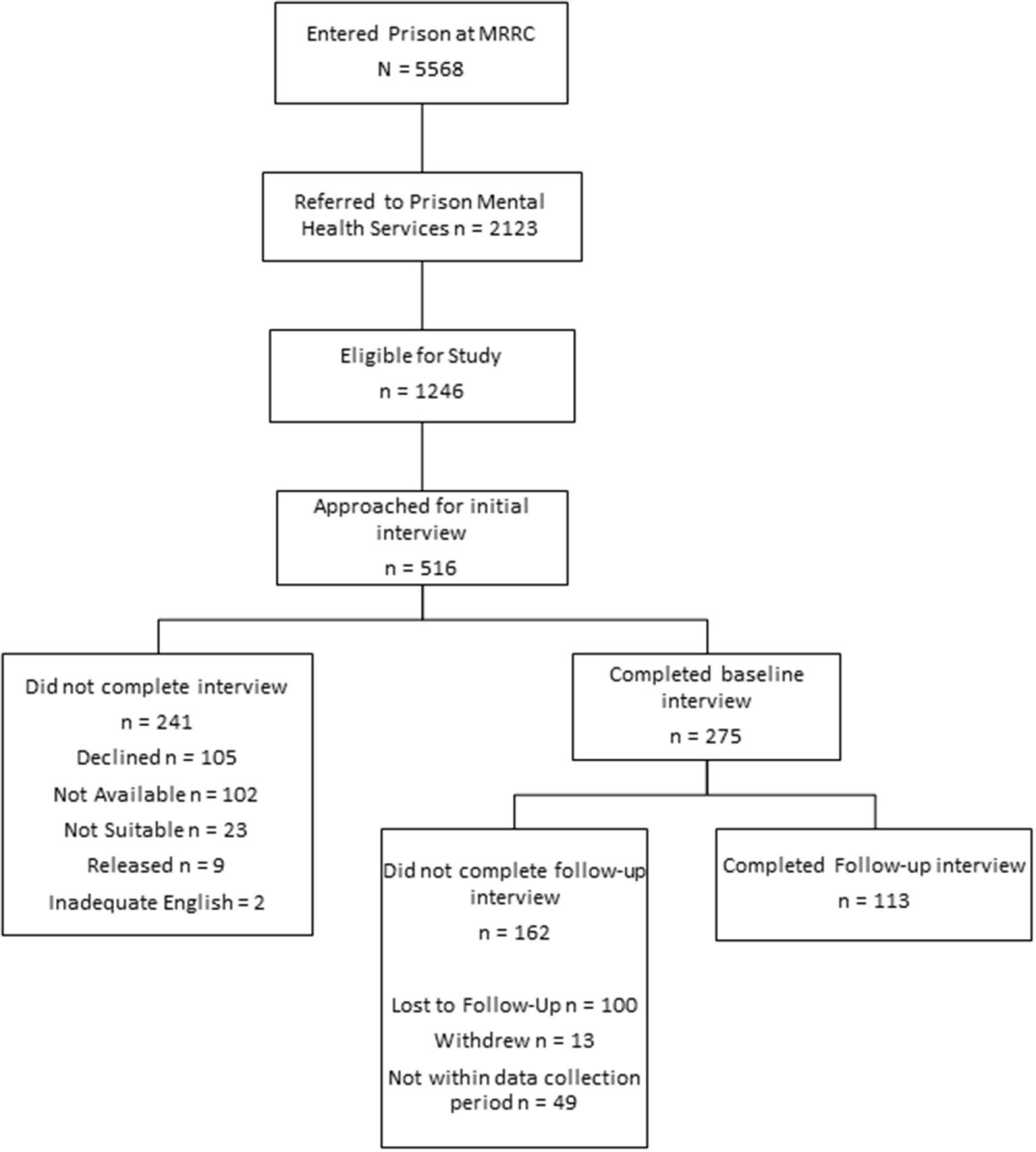
Participant recruitment process.

During the recruitment period, 5,568 individuals entered prison at the MRRC of which 2,123 (38%) were referred to prison mental health services (Figure 1). Of these, 1,283 (60%) were charged with minor (i.e., non-indictable) offenses or had been sentenced to a period of imprisonment of under 6 months. A small number of these (37; 3%) were excluded on the grounds of having previously been approached, leaving 1,246 eligible (59%). Time and resource constraints (such as limited daily ‘out of cell' hours in which to conduct interviews, limited dedicated interview spaces within the prison, and frequent prison lockdowns) allowed researchers to attempt to approach 516 (41%) of those eligible during the recruitment period. Of these, 241 (47%) were not able to be interviewed: 105 (44%) declined, 102 (42%) were unavailable (e.g., at court or other appointments), 23 (10%) were unsuitable (e.g., due to incapacity arising from their mental state or risks arising from their behavioral presentation), nine (4%) were released prior to approach, and two (<1%) were unable to be interviewed due to inadequate English proficiency. In total, 275 of the individuals approached (53%) consented to and completed the baseline data collection interview.

Follow-up interviews were conducted with 113 participants (41.1%) at 3 months post-release. Of the remaining individuals who were not interviewed at followed up (*n* = 162; 58.9%), 100 were lost to follow-up, 13 declined to continue with the study and 49 were either released after the follow-up data collection period ended (September 2020) or had not reached 3 months post-release by the end of the period.

### Sample description

#### Socio-demographic and criminal justice characteristics

The sociodemographic and criminal justice characteristics of the total sample at baseline interview are presented in Table 1. Participants' ages ranged from 18 to 76 years [median = 37, Interquartile Range (IQR) = 28–48]. One quarter (25.8%) identified as Aboriginal and/or Torres Strait Islander. Over half of the sample (58.9%) reported being single. A third (33.5%) had an educational attainment below tenth grade level (i.e., end of secondary schooling) and three quarters (74.9%) were unemployed at the time when they were charged with the current offense/s. Nearly one in five reported being homeless or in unstable accommodation at the time they were charged. Nearly half (47.2%) reported a family history of mental illness.

**Table 1:** Sociodemographic and criminal justice characteristics of the interviewed sample at baseline.

**Sample characteristics**	**Total sample (*N* = 275)**
**Sociodemographic**
Median age (years)	37 [IQR 28–43]
Aboriginal and/or Torres Strait Islander	71 (25.8%)
Marital status
Single	162 (58.9%)
Married/defacto/partnered	88 (32.0%)
Divorced/separated/widowed	24 (8.7%)
Highest level of education < year 10	92 (33.5%)
Unemployed	206 (74.9%)
Homeless/unstable accommodation	52 (18.9%)
Family history of mental illness	130 (47.3%)
**Criminal justice**
Legal status
Remand	242 (88.0%)
Sentenced	33 (12.0%)
Charges include violence	114 (41.5%)
First time in custody	45 (16.4%)

The majority of the sample were on remand (88%) with a small proportion (12%) having already been sentenced to a period of <6 months, and 41.5% of the sample had been charged with a violent offense. The majority had been incarcerated before, with this being the first time in prison for only 16.4% of the sample.

#### Clinical characteristics and previous community mental health team contact

By definition all participants (*n* = 275) had been referred to prison mental health services on reception screening. Approximately a third (30.5%) were also referred to drug and alcohol services (Table 2). The majority of the sample (89.8%) had a self-reported history of being prescribed psychiatric medication and 61.8% reported a history of hospital admissions for mental illness. Review of individual prison health records revealed that the majority (97.5%) had received a mental health diagnosis from a mental health professional in their lifetime; just over half (54.2%) had been diagnosed with a serious mental illness (schizophrenia, psychotic disorders, bipolar disorder) and 65.5% had received a common mental disorder diagnosis (depression, anxiety). In terms of self-reported symptoms, 94.5% reported experiencing at least one mental health symptom in the last month. Two thirds (66.2%) reported psychotic symptoms in the last month (of whom 60.4% had a serious mental illness diagnosis, and 39.6% had not); and 85.1% reported having experienced lifetime psychotic symptoms.

**Table 2:** Clinical characteristics of the interviewed sample at baseline.

**Clinical characteristics**	**Total sample**
	**(*N* = 275)**
**Referrals from reception**
Mental health	275 (100.0%)
Drug and alcohol	84 (30.5%)
Risk intervention team	20 (7.3%)
**Mental Health**
Ever prescribed psychiatric medication	247 (89.8%)
History of mental health hospital admissions	170 (61.8%)
**Ever diagnosed with:**
Schizophrenia spectrum/psychotic disorder	127 (46.2%)
Depressive disorder	159 (57.8)
Anxiety disorder	141 (51.3%)
Bipolar disorder	45 (16.4%)
Trauma-related disorder	57 (20.7)
Personality disorder/traits	80 (29.1%)
Attention deficit/hyperactivity disorder	44 (16.0%)
Substance/alcohol use disorder	159 (57.8%)
Any mental health diagnosis	268 (97.5%)
Serious mental illness (SMI) diagnosis	149 (54.2%)
Common mental disorder diagnosis	180 (65.5%)
Any recent (last month) mental health symptoms	260 (94.5%)
Any history of psychotic symptoms	234 (85.1%)
Any recent (last month) psychotic symptoms	182 (66.2%)
SMI diagnosis	110 (60.4%)
No SMI diagnosis	72 (39.6%)
**Suicide and self-harm**
Any history of self-harm (excluding suicide attempts)	90 (32.7%)
Any history of suicide attempts	108 (39.3%)
Any history of self-harm or suicide attempts	140 (50.9%)
Suicidal thoughts or ideation in past week	79 (28.7%)
**Substance use**
Consumed alcohol in last 3 months	178 (64.7%)
Illicit drug use in last 3 months	220 (80%)
Daily drug use in last 3 months	147 (53.5%)
**Any drug use in last 3 months (*****n*** **=** **220)**
Pills (non-prescribed medication)	60 (27.3%)
Heroin	69 (31.4%)
Cannabis	150 (68.2%)
Methamphetamine	166 (75.5%)
Cocaine	36 (16.4%)
Ecstasy	23 (10.5%)
GHB	34 (15.5%)
LSD/hallucinogens	13 (5.9%)
Other drugs	6 (2.7%)
Intoxicated at time of offense (drugs or alcohol)	162 (58.9%)
**Community mental health care**
Any past contact with CMHT	69 (25.1%)
Linked in with CMHT prior to prison	34 (12.4%)

A small proportion of the total sample (7.3%) was referred from reception screening to the Risk Intervention Team for assessment and management of acute suicide and self-harm risk. Half of the sample (50.9%) had a history of self-harm or suicide attempts and over a quarter (28.7%) reported suicidal thoughts or ideation in the past week.

The majority of the sample (80%) had engaged in illicit drug use in the 3 months prior to prison and, of these, 66.4% reported using daily (53.5% of the total sample). Nearly two-thirds (64.7%) reported consuming alcohol in the 3 months prior.

A quarter of the total sample (25.1%) reported having past contact with a community mental health team (CMHT) and 12.4% were linked in with a team at the time of coming into prison.

#### Comparison between those completing the post-release 3-month interview and those lost to follow-up

No significant differences were found between those who were followed up by the research team at 3 months (*n* = 113) and those not followed up (i.e., those either lost to follow-up or those who had not reached the 3-month post-release point before the end of the data collection period; *n* = 149), in terms of key sociodemographic or criminal justice variables (Table 3). Those who were not followed up were significantly more likely to have reported a history of psychotic symptoms at baseline interview ($\chi_{\left(1\right)}^{2}$ = 3.97, *p* < 0.05) but no more likely to have a diagnosis of serious mental illness or to have reported recent psychotic symptoms. There was also no significant difference between the groups in terms of having been prescribed medication for their mental health, history of hospitalization, suicide/self-harm history, drug use (any use of drugs in the 3 months prior to prison, or daily use), prior contact with community mental health services, or referral for mental health care at release.

**Table 3:** Comparison of baseline characteristics of participants followed up by the research team at 3 months post release with those lost to follow-up.

**Characteristics**	**Followed up (*n* = 113)**	**Not followed up (*n* = 149)**	**χ^2^ (*df*)**
**Sociodemographic/criminal justice**
Aboriginal and/or Torres Strait Islander	26 (23.0%)	43 (28.9%)	1.13 (1)
Highest level of education < year 10	35 (31.0%)	54 (36.2%)	0.80 (1)
Unemployed	85 (75.2%)	111 (74.5%)	0.02 (1)
Homeless/unstable accommodation	22 (19.5%)	31 (20.9%)	0.09 (1)
Charges include violence	52 (46.0%)	57 (38.3%)	1.59 (1)
First time in custody	21 (18.6%)	21 (14.1%)	0.96 (1)
**Clinical**
Ever prescribed psychiatric medication	102 (90.3%)	134 (89.9%)	0.01 (1)
History of mental health hospital admissions	72 (64.3%)	90 (60.8%)	0.33 (1)
Serious mental illness diagnosis	57 (50.4%)	84 (56.4%)	0.91 (1)
Any history of psychotic symptoms	90 (79.6%)	132 (88.6%)	3.97 (1)^*^
Any recent (last month) psychotic symptoms	74 (65.5%)	98 (65.8%)	0.00 (1)
Any history of self-harm or suicide attempts	60 (53.6%)	76 (51.0%)	0.17 (1)
Illicit drug use in 3 months prior to custody	91 (80.5%)	117 (78.5%)	0.16 (1)
Daily drug use in 3 months prior to custody	60 (53.1%)	79 (53.0%)	0.00 (1)
**Community mental health care**
Any past contact with CMHT	25 (22.5%)	43 (29.9%)	1.73 (1)
Linked in with CMHT prior to prison	12 (10.8%)	20 (13.6%)	0.46 (1)
Referral for MH care at release	13 (11.5%)	21 (14.5%)	0.49 (1)

* p < 0.05.

### Mental health contacts during incarceration and circumstances of release

The majority (85.5%) of the total sample had contact with the prison mental health service during their period of incarceration. Three quarters of the sample (74.5%) were seen within a week of being referred and 80.7% had been seen within the first month. A small percentage (4.8%) waited for more than a month to be seen while 14.5% were released before they had any mental health service contact. Those who did not have contact with mental health services before release spent a median of 16 days in custody (IQR = 3–49).

The majority of the sample spent <6 months in prison following the baseline interview (77.5%). More than one in five (22.2%) were released and reincarcerated at least once in this time. Within 6 months of the baseline interview, 88.7% had seen a prison mental health professional at least once, most commonly a mental health nurse (88.0%); 38.2% had seen a psychiatrist. Over half (57.8%) were seen more than once during this period. The median number of mental health contacts was 2 (IQR = 1–4).

Just over half (55%) of those released during the study period (*n* = 271), had a set date for release, either to parole or due to their sentence coming to an end. The other half (45%) had uncertainty around release, and were released from court, due to either being granted bail, given an alternative to a custodial sentence (e.g., community corrections order, other penalty such as a fine imposed), having charges dismissed or being disposed of under the Mental Health (Forensic Provisions) Act 1990 (22).

### Referral to mental health services at time of release

**Figure 2:**
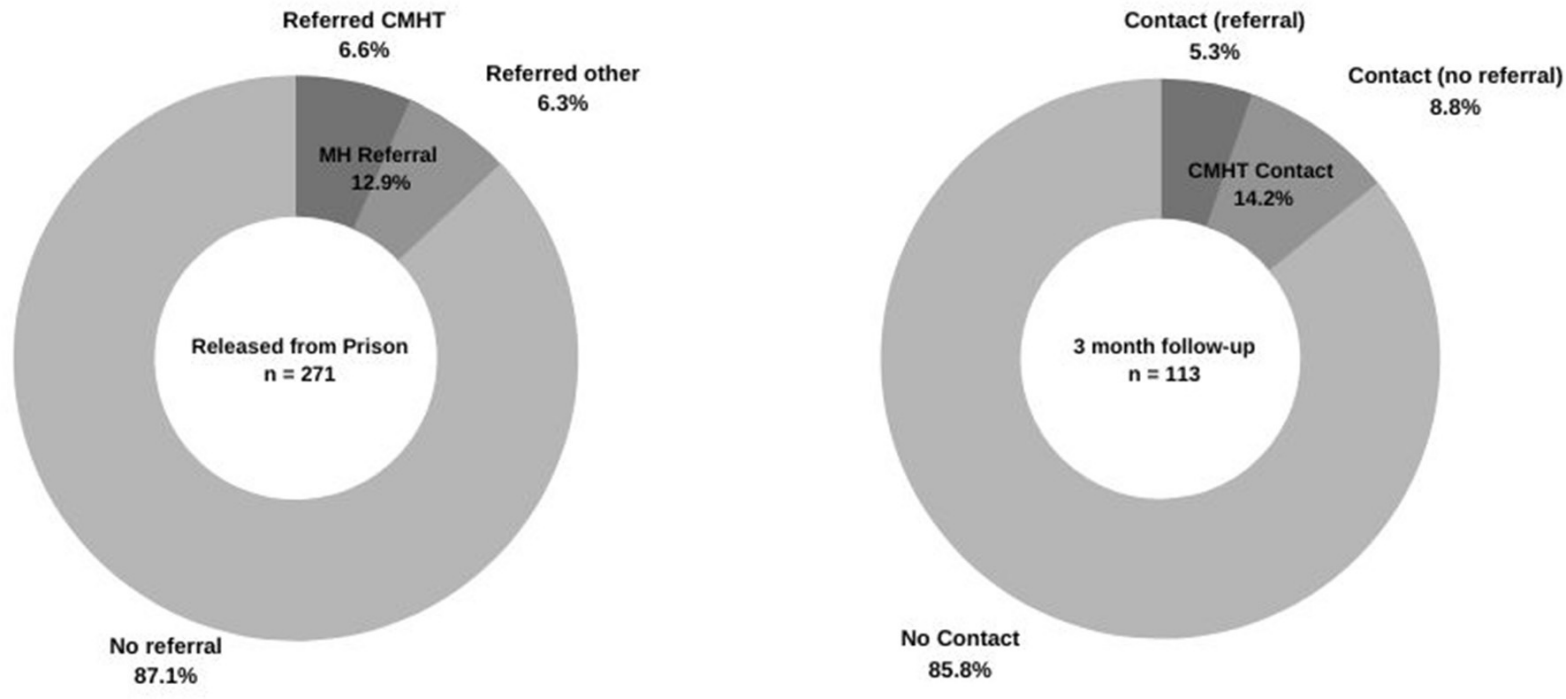
Proportion of released participants who received a referral for mental health care, and proportion of those interviewed at follow-up who had contact with a Community Mental Health Team.

Of all those released, 18 (6.6%) were referred by the prison mental health service to a community mental health team at time of release and 17 (6.3%) were either referred elsewhere for mental health care (e.g., GP or other primary care provider, *n* = 10), referred by Parole (*n* = 1), or released under a mental health order (Section Sample description or Section Mental health contacts during incarceration and circumstances of release of the Mental Health Act), (*n* = 6) meaning that some kind of mental health care was mandated (Figure 2). In total, 35 (12.9%) of participants in our sample received a referral or order for mental health care post-release.

While there was no significant difference in the sociodemographic profile of those referred vs. those not referred (Table 4), participants with violent charges were twice as likely to receive a referral or order for mental health care (OR = 2.13, 95% CI = 1.04, 4.36). Those referred were six times more likely to have a diagnosis of serious mental illness (OR = 6.00, 95% CI = 2.25, 16.00), and more than two and half times more likely to have reported recent psychotic symptoms at time of prison entry (OR = 2.77, 95% CI = 1.11, 6.94). They were also four times more likely to have had past contact with a Community Mental Health Team (OR = 4.11, 95% CI = 1.94, 8.71) and around three and a half times more likely to have been linked in with a team at the time of entering custody (OR = 3.63, 95% CI = 1.55, 8.49).

**Table 4:** Comparison of characteristics of participants who received a referral or order for mental health care post-release, with those not referred.

**Characteristics**	**Received referral (*n* = 35)**	**No referral (*n* = 236)**	**χ^2^ (*df*)**	**Odds ratio [95% CI]**
**Sociodemographic/criminal justice**
Aboriginal and/or Torres Strait Islander	10 (28.6%)	60 (25.4%)	0.16 (1)	1.17 [0.53, 2.59]
Highest level of education < year 10	11 (31.4%)	80 (33.9%)	0.01 (1)	0.89 [0.42, 1.92]
Unemployed	30 (85.7%)	174 (73.7%)	2.35 (1)	2.14 [0.79, 5.75]
Homeless/unstable accommodation	10 (28.6%)	48 (20.4%)	0.20 (1)	1.56 [0.70, 3.47]
Charges include violence	20 (57.1%)	91 (38.6%)	4.35 (1)^*^	2.13 [1.04, 4.36]
First time in custody	2 (5.7%)	40 (16.9%)	2.94 (1)	0.30 [0.07, 1.29]
**Clinical**
Ever prescribed psychiatric medication	32 (91.4%)	212 (89.8%)	0.09 (1)	1.21 [0.34, 4.24]
History of mental health hospital admissions	27 (77.1%)	140 (60.1%)	3.77 (1)	2.24 [0.98, 5.15]
Serious Mental Illness diagnosis	30 (85.7%)	118 (50.0%)	15.68 (1)^**^	6.00 [2.25, 16.00]
Any history of psychotic symptoms	33 (94.3%)	197 (83.5%)	2.77 (1)	3.27 [0.75, 14.18]
Any recent (last month) psychotic symptoms	29 (82.9%)	150 (63.6%)	5.06 (1)^*^	2.77 [1.11, 6.94]
Any history of self-harm or suicide attempts	22 (62.9%)	116 (49.4%)	2.22 (1)	1.74 [0.84, 3.61]
Illicit drug use in last 3 months	32 (91.4%)	185 (78.4%)	3.25 (1)	2.94 [0.87, 9.99]
Daily drug use in last 3 months	21 (60.0%)	124 (52.5%)	0.68 (1)	1.36 [0.66, 2.79]
**Community mental health care**
Any past contact with CMHT	18 (54.5%)	52 (22.6%)	15.07 (1)^**^	4.11 [1.94, 8.71]
Linked in with CMHT prior to prison	10 (29.4%)	24 (10.3%)	9.75 (1)^*^	3.63 [1.55, 8.49]

* p < 0.05.

** p < 0.001.

### Post-release community mental health contact

Of those who engaged in a follow-up interview 3 months post-release (*n* = 113, Figure 2), only 16 (14.2%) had been in contact with a community mental health team, of whom five had been referred to a team at time of release, one had received a referral to another mental health care provider, and 10 had received no referral for mental health follow-up.

### Post-release clinical and reoffending outcomes

Of those followed up by the research team, 46 (40.7%) reported receiving therapy or counseling in the 3 months since release and 74 (65.5%) reported currently taking medication for mental health. Around three quarters (73.5%) reported experiencing at least one mental health symptom from the screening tool within the previous month, compared with the 94.5% who reported at least one symptom at baseline; and 43.4% reported at least one recent psychotic symptom at follow-up as compared with 66.2% at baseline. Around half of the sample (54%) reported consuming alcohol in the 3 months post release compared with 64.7% at baseline, and a similar proportion (48.7%) reported use of illicit drugs compared to the 80% reported at baseline. Nearly one in five (19.5%) reported using illicit drugs daily, less than the 66.4% reported at baseline.

Of those who had been released, reached 3 months post-release within the data collection period, and who had agreed to continue their participation in the study (*n* = 255), a third (32.9%, *n* = 84) had received further charges including for technical violations (i.e., breach of orders) and 8.2% (*n* = 21) had received further charges for violence. One-fifth (21.6%, *n* = 55) had been reincarcerated within 3 months. Those reincarcerated within 3 months were more likely to be Aboriginal and/or Torres Strait Islander (OR = 2.36, 95% CI = 1.25, 4.46), to have been charged with non-violent offenses at time of baseline interview (OR = 1.95, 95% CI = 1.03, 3.73) and to have experienced previous incarceration (OR = 12.26, 95% CI = 1.64, 91.48), but no more likely to have been homeless or unemployed prior to custody, or to have an education level of less than year 10 (Table 5). There was no significant association found between clinical factors at baseline (psychiatric medication, psychiatric admissions, serious mental illness diagnosis, or psychotic symptoms) and reincarceration within 3 months. There was also no significant association found between previous contact with a community mental health team, mental health referral at release, history of self-harm or suicide attempts, or drug use, and reincarceration within 3 months.

**Table 5:** Comparison of baseline sociodemographic, criminal justice and clinical characteristics of participants reincarcerated within 3 months of release, with those not reincarcerated.

**Characteristics**	**Reincarcerated (*n* = 55)**	**Not reincarcerated (*n* = 200)**	**χ^2^ (*df*)**	**Odds ratio [95% CI]**
**Sociodemographic/criminal justice**
Aboriginal and/or Torres Strait Islander	22 (40.0%)	44 (22.0%)	7.29 (1)^*^	2.36 [1.25, 4.46]
Highest level of education < year 10	25 (45.5%)	63 (31.5%)	3.72 (1)	1.81 [0.99, 3.33]
Unemployed	43 (78.2%)	151 (75.5%)	0.17 (1)	1.16 [0.57, 2.38]
Homeless/unstable accommodation	10 (18.2%)	42 (21.1%)	0.23 (1)	0.83 [0.39, 1.79]
Non-violent charges	39 (70.9%)	111 (55.5%)	4.23 (1)^*^	1.95 [1.03, 3.73]
Previous incarceration	54 (98.2%)	163 (81.5%)	9.47 (1)^*^	12.26 [1.64, 91.48]
**Clinical**
Ever prescribed psychiatric medication	48 (87.3%)	182 (91.0%)	0.68 (1)	0.68 [0.27, 1.72]
History of mental health hospital admissions	32 (59.3%)	125 (62.8%)	0.23 (1)	0.86 [0.47, 1.59]
Serious mental illness diagnosis	31 (56.4%)	107 (53.5%)	0.14 (1)	1.12 [0.62, 2.05]
Any history of psychotic symptoms	49 (89.1%)	166 (83.0%)	1.21 (1)	1.67 [0.66, 4.22]
Any recent (last month) psychotic symptoms	33 (60.0%)	133 (66.5%)	0.80 (1)	0.76 [0.41, 1.40]
Any history of self-harm or suicide attempts	24 (43.6%)	107 (53.8%)	1.77 (1)	0.67 [0.37, 1.22]
Illicit drug use in 3 months prior to custody	45 (81.8%)	158 (79.0%)	0.21 (1)	1.20 [0.56, 2.57]
Daily drug use in 3 months prior to custody	31 (56.4%)	105 (52.5%)	0.26 (1)	1.17 [0.64, 2.13]
**Community mental health variables**
Any past contact with CMHT	15 (28.8%)	53 (27.0%)	0.07 (1)	1.09 [0.55, 2.15]
Linked in with CMHT prior to prison	6 (11.3%)	26 (13.1%)	0.12 (1)	0.85 [0.33, 2.17]
Referral for MH care at release	8 (14.5%)	25 (12.5%)	0.16 (1)	1.19 [0.51, 2.81]

* p < 0.05.

## Discussion

In this Australian study of a sample of men spending short periods of time in prison and presenting on reception with significant mental health needs, very few were released from prison with evidence of mental health discharge planning. At 3 months post-release, the proportion experiencing significant mental health symptoms was lower than at baseline, but still high, and few reported having any contact with community mental health services since release. These findings support the need for a specific focus on developing mental health services and interventions for those spending relatively brief but disruptive periods in prison, a group often not considered in prison mental health research and service development efforts. While diverting these individuals from the criminal justice system where possible should remain a priority, improving continuity of care in the prison to community transition period is also important for addressing the overall burden of mental illness and unmet need amongst those with mental illness who have contact with the criminal justice system.

### Main findings

Our sample was broadly comparable with surveys of prisoners conducted nationally and in NSW in terms of sociodemographic characteristics (2, 23). Our findings reflect that those in prison are amongst the most disadvantaged populations, with high rates of homelessness, unemployment, and previous incarcerations. A quarter of our sample identified as Aboriginal and/or Torres Strait Islander, highlighting the ongoing over-representation in Australian prisons of this group, who make up around 3.3% of the general population (24).

In our sample of individuals who had been referred to mental health services upon reception into prison, the majority reported that they had previously received a mental health diagnosis and been prescribed psychiatric medication. Around half had a serious mental illness diagnosis; the majority reported having ever experienced psychotic symptoms and two thirds of the sample reported experiencing psychotic symptoms in the past month. Despite high levels of psychiatric diagnosis and symptomatology, only a quarter of the sample had ever had contact with community mental health services and only a little over one in 10 were linked in with a team at the time of entering prison, likely reflecting the high level of unmet mental health need that this group experience even when in the community.

The majority of participants in our sample were seen by a mental health professional at least once during their time in prison, however around one in seven were released before having any contact with mental health services, indicating that a significant proportion of those identified as having mental health needs at reception screening are not receiving any mental health care during their short periods in custody. Evidence of planning for supporting continuity of mental health care following discharge was lacking: only around one in eight in our sample were released with a referral to a mental health care provider in the community or a mental health treatment order. In addition to the relatively short period of time spent by participants in prison (<6 months for more than three quarters of the sample), a key factor in the lack of care planning prior to release is likely to have been the unpredictability of release; almost half of the sample were released directly from court without warning. Discharge planning and effective handover of care is likely to be very difficult for prison mental health services to manage under these circumstances. Qualitative research conducted in NSW exploring the experiences of those involved in supporting the transition of individuals with serious mental illness from prison to the community (25) supports this, with prison health staff reporting difficulties in obtaining information about prisoner release. This suggests that strategies to improve communication and information sharing between health and court/correctional systems may lead to an increased rate of referral to community mental health services which would contribute to continuity of care for this group.

Another difficulty associated with uncertainty around release dates is securing stable accommodation at short notice. Almost one in five of our sample reported being homeless or in unstable accommodation at the time of prison entry, and we can surmise that at least this proportion, if not higher, would be released without having stable accommodation secured. As individuals are linked with community mental health teams based on geographical location, not having an address on release impacts the ability of prison staff to make a referral to a community mental health team. Even where a referral to a community mental health team is made, an individual with insecure housing or an itinerant lifestyle is likely to experience difficulties maintaining ongoing engagement with a team, and the ability of teams to locate patients in the community (if they do not self-present for care) is likely to be impacted. This suggests that discharge planning, including securing post-release accommodation, needs to occur very soon after prison entry for individuals identified as likely to spend a short period in prison, and that the provision of secure accommodation options for individuals exiting prison needs to be prioritized.

Low rates of referral and uncertainty around discharge considered, it is perhaps unsurprising that at 3 months post-release only 14.2% of those in our sample who were followed up by the research team had been in contact with a community mental health team. Less than half of these individuals, however, had been referred to a team at time of release from prison, indicating that they either sought out their own mental health care after release or were referred by other means, such as through parole or primary care providers. Around 40% of those interviewed by the research team reported that they had received therapy or counseling in the 3 months since release which reinforces that individuals are receiving mental health services independent to those provided by community mental health teams. The mental health needs of those exiting prison are clearly high; rates of emergency department attendance are elevated in this group (26), as are inpatient hospital admissions (27). However, those with mental health difficulties and criminal justice involvement commonly experience difficulties in obtaining mental health care in the community, often due to the stigma associated with their offending behavior (28). Community mental health facilities are often ill-equipped to manage the complex needs of these patients (29) and those recently released from prison may experience lengthy delays or difficulty in securing psychiatric appointments (30), highlighting the need for development of specialized processes and services for these individuals in the community.

A significant proportion of participants continued to experience mental health symptoms at 3 months post-release, although less than at baseline. This may reflect the fact that levels of psychological distress and psychiatric symptomatology are often highest at prison entry (31, 32). Additionally, it is possible that there is an association between level of mental illness and loss to follow-up in that those with more severe symptoms are less likely to be able to be contacted for follow-up. While this study did not find differences between those followed up and those who were not in terms of sociodemographic or criminal justice characteristics, those who reported a history of psychotic symptoms were less likely to have been followed up by the researchers. Similarly, the decrease in self-reported recent drug use from baseline to follow-up may be explained by biased attrition rates.

Those who cycle through prison for brief periods are likely to face the same difficulties in obtaining treatment for drug and alcohol use as they are in obtaining mental health care which is evidenced by our finding that despite 80% of our sample reporting illicit drug use in the 3 months prior to prison (and two-thirds of these using daily), less than a third were referred to the prison drug and alcohol service at reception. Those with comorbid psychiatric and substance use disorder are at higher risk of multiple short-term periods of incarceration than those with mental illness or substance use disorders alone (33) which suggests that approaches to treatment and planning for discharge must address both problems if substantial reductions in levels of incarceration, mental illness and problematic substance use are to be realized.

In terms of reoffending outcomes, around one third of our sample had been charged with further offenses in the 3-month post-release period and one-fifth had been reincarcerated. While there are no published data on local population re-offending rates within 3 months of release from prison, it is well established that almost half of all individuals released from prison in NSW return to prison within 2 years (34). The proportion of the study sample who reoffended within such a short period following release is concerning and reinforces the improbability of short-term incarceration serving a rehabilitative function, while instead causing substantial disruption to healthcare continuity, housing stability, employment stability, and social/cultural connectivity.

### Strengths and limitations

Individuals with mental illness who experience short, frequent periods of incarceration are an understudied group; to our knowledge this is the only study to identify these individuals at the point of reception into prison and prospectively examine patterns of mental health contact in prison and mental health and criminal justice outcomes post-release. As expected, conducting research focused on this group was challenging, with nearly half of those approached for participation either declining to participate, being unavailable due to other appointments, or unsuitable due to mental state or behavioral risk. Additionally, the same factors that impact on mental healthcare continuity also affect rates of longitudinal research engagement. We were only able to complete follow-up interviews in the community with around 40% of those recruited and thus there may be biases operating in relation to our post-release findings. For example, our finding that those who were unable to be followed up were more likely to have reported a history of psychotic symptoms indicates that we may not have captured for follow-up those at the more severe end of the mental illness spectrum. Further research into mental health outcomes for this group obtained by methods other than participant self-report at follow-up, such as data linkage, may provide a clearer picture of rates of mental health service use post-release from prison.

In addition to the potential biases resulting from differential attrition, loss to follow-up had an impact on the statistical power available to examine a number of associations at the post-release point. For example, no formal analyses could be undertaken to determine if mental health referral had any impact on rates of mental health contact or reoffending in the 3 months post-release.

Finally, this study examines continuity of mental health care for only a subgroup of individuals who experience incarceration and does not provide evidence for the overall state of continuity of care between prison and the community, including for those spending longer periods in prison who may have more opportunity for discharge planning. The current sample contained men only and future research would benefit from the inclusion of women, who are known to experience a higher burden of mental ill health in prison (35). Furthermore, this study considers continuity of care in only one jurisdiction: NSW, Australia, and given the differing justice and health care systems between states and internationally, the generalizability of these results may be limited. Further research is required across jurisdictions to examine continuity of mental health care between prison and the community, and its impact on clinical and reoffending outcomes.

## Conclusion

Achieving continuity of mental health care between prison and the community is challenging, particularly for those who experience frequent short periods of incarceration. Our study demonstrates that many are released with little opportunity for discharge planning and rates of community mental health contact in the early post-release period are low. While there is emerging evidence for the effectiveness of transition programs in increasing mental health contacts in the community post-release (12), these programs are not widely available, and given the short period many spend in prison and the proportion of those with uncertainty about release timing, many would not be eligible for such programs. It is paradoxical that those who are arguably most in need of intensive support and continuity of care are the least likely to receive it, resulting in a cycle of instability and re-incarceration.

Early identification, provision of assertive mental health treatment, and early discharge planning for individuals likely to fall into this group is essential if we are to reduce the overall burden of mental illness in prison and its associated adversity. A targeted approach to continuity of care involving both community and custodial mental health services, as well as the court system, is required. Considering the harmful effects of incarceration and the barriers to continuity of care between prison and community that have been outlined in this paper, consideration must be given to wider implementation of alternatives to custody for those with mental illness who commit minor offenses, and for whom a brief period in prison will only exacerbate the mental health and social problems that have likely contributed to their criminal justice system involvement.

## Data availability statement

Access to the dataset will require approval from the relevant ethics committees outlined above as well as Justice Health and Forensic Mental Health Network as data custodians. Requests to access the datasets should be directed at: christie.browne@health.nsw.gov.au.

## Ethics statement

The studies involving human participants were reviewed and approved by Justice Health and Forensic Mental Health Network Human Research Ethics Committee (Ref: G185/14) NSW Aboriginal Health and Medical Research Council Ethics Committee (Ref: 1137/15) Corrective Services New South Wales Ethics Committee (Ref: D16/139081) Western Sydney Local Health District Human Research Ethics Committee (Ref: LNR/18/WMEAD/461). The patients/participants provided their written informed consent to participate in this study.

## Author contributions

CB and KD conducted the analysis of the results and writing of the manuscript. All authors have reviewed and provided input into the manuscript and contributed to the design and implementation of the research.

## Funding

This research was supported by National Health and Medical Research Council (NHMRC) Grant: The Australian Centre for Research Excellence in Offender Health (GNT1057492) and NHMRC Investigator Grant: Improving the Mental Health of People in Contact, or at Risk of Contact, with the Criminal Justice System (APP1175408). New South Wales Ministry of Health Translational Research Grant: Improving the mental health of released prisoners: an RCT of a Critical Time intervention characterized by early and individualized care planning, and Justice Health and Forensic Mental Health Network (JHFMHN) provided in-kind support of JHFMHN clinicians and researchers involved in the project.

## Conflict of interest

The authors declare that the research was conducted in the absence of any commercial or financial relationships that could be construed as a potential conflict of interest.

## Publisher's note

All claims expressed in this article are solely those of the authors and do not necessarily represent those of their affiliated organizations, or those of the publisher, the editors and the reviewers. Any product that may be evaluated in this article, or claim that may be made by its manufacturer, is not guaranteed or endorsed by the publisher.

## References

[1] Fazel S Seewald K. Severe mental illness in 33, 588 prisoners worldwide: systematic review and meta-regression analysis. Br J Psychiatry. (2012) 200:364–73. 10.1192/bjp.bp.111.096370 22550330

[2] Australian Institute of Health and Welfare. The health of Australia's prisoners 2018. Cat. no. PHE 246. Canberra: AIHW (2019).

[3] Australian Bureau of Statistics. National Health Survey: First Results, 2017-18. Cat. no. 4364, 0.55.001. Canberra: ABS (2018).

[4] Butler T Andrews G Allnutt S Sakashita C Smith NE Basson J. Mental disorders in Australian prisoners: a comparison with a community sample. Aust N Z J Psychiatry. (2006) 40:272–6. 10.1080/j.1440-1614.2006.01785.x 16476156

[5] Baillargeon J Binswanger IA Penn JV Williams BA Murray OJ. Psychiatric disorders and repeat incarcerations: the revolving prison door. Am J Psychiatry. (2009) 166:103–9. 10.1176/appi.ajp.2008.08030416 19047321

[6] Jones RM Manetsch M Gerritsen C Simpson AI. Patterns and predictors of reincarceration among prisoners with serious mental illness: a cohort study: modèles et prédicteurs de réincarcération chez les prisonniers souffrant de maladie mentale grave: une étude de cohorte. Can J Psychiatry. (2021) 66:560–8. 10.1177/0706743720970829 33155829 PMC8138736

[7] Davidson F Clugston B Perrin M Williams M Heffernan E Kinner SA. Mapping the prison mental health service workforce in Australia. Australas Psychiatry. (2020) 28:442–7. 10.1177/1039856219891525 31868515

[8] Australian Bureau of Statistics. Criminal Courts, Australia, 2020. (2020). Available online at: https://www.abs.gov.au/statistics/people/crime-and-justice/criminal-courts-australia/latest-release#key-statistics (accessed February 16, 2022).

[9] Baillargeon J Hoge SK Penn JV. Addressing the challenge of community reentry among released inmates with serious mental illness. Am J Community Psychol. (2010) 46:361–75. 10.1007/s10464-010-9345-6 20865315

[10] Hawthorne WB Folsom DP Sommerfeld DH Lanouette NM Lewis M Aarons GA. Incarceration among adults who are in the public mental health system: Rates, risk factors, and short-term outcomes. Psychiatr Ser. (2012) 63:26–32. 10.1176/appi.ps.201000505 22227756

[11] Lennox C Senior J King C Hassan L Clayton R Thornicroft G. The management of released prisoners with severe and enduring mental illness. J Forens Psychiatry Psychol. (2012) 23:67–75. 10.1080/14789949.2011.634921

[12] Hopkin G Evans-Lacko S Forrester A Shaw J Thornicroft G. Interventions at the transition from prison to the community for prisoners with mental illness: a systematic review. Adm Policy Ment Health. (2018) 45:623–34. 10.1007/s10488-018-0848-z 29362981 PMC5999162

[13] Hwang YI Albalawi O Adily A Hudson M Wand H Kariminia A. Disengagement from mental health treatment and re-offending in those with psychosis: a multi-state model of linked data. Soc Psychiatry Psychiatr Epidemiol. (2020) 55:1639–48. 10.1007/s00127-020-01873-1 32390094

[14] Domino ME Gertner A Grabert B Cuddeback GS Childers T Morrissey JP. Do timely mental health services reduce re-incarceration among prison releasees with severe mental illness? Health Serv Res. (2019) 54:592–602. 10.1111/1475-6773.13128 30829406 PMC6505414

[15] Green B Denton M Heffernan E Russell B Stapleton L Waterson E. From custody to community: outcomes of community-based support for mentally ill prisoners. Psychiatr Psychol Law. (2016) 23:798–808. 10.1080/13218719.2016.1152926

[16] Thomas EG Spittal MJ Heffernan EB Taxman FS Alati R Kinner SA. Trajectories of psychological distress after prison release: implications for mental health service need in ex-prisoners. Psychol Med. (2016) 46:611–21. 10.1017/S0033291715002123 26549475

[17] Borschmann R Young JT Moran P Spittal MJ Heffernan E Mok K. Ambulance attendances resulting from self-harm after release from prison: a prospective data linkage study. Soc Psychiatry Psychiatr Epidemiol. (2017) 52:1295–305. 10.1007/s00127-017-1383-z 28389689

[18] Kariminia A Law MG Butler TG Levy MH Corben SP Kaldor JM. Suicide risk among recently released prisoners in New South Wales, Australia. Med J Aust. (2007) 187:387–90. 10.5694/j.1326-5377.2007.tb01307.x 17908000

[19] Forsyth SJ Carroll M Lennox N Kinner SA. Incidence and risk factors for mortality after release from prison in Australia: a prospective cohort study. Addiction. (2018) 113:937–45. 10.1111/add.14106 29154395

[20] Kariminia A Law MG Butler TG Corben SP Levy MH Kaldor JM. Factors associated with mortality in a cohort of Australian prisoners. Eur J Epidemiol. (2007) 22:417–28. 10.1007/s10654-007-9134-1 17668280

[21] Kinner SA. Continuity of health impairment and substance misuse among adult prisoners in Queensland, Australia. Int J Prison Health. (2006) 2:101–13. 10.1080/17449200600935711

[22] Mental Health (Forensic Provisions) Act (1990). Available online at: https://legislation.nsw.gov.au/view/html/repealed/current/act-1990-010 (accessed April 26, 2022).

[23] Justice Health and Forensic Mental Health Network. 2015 Network Patient Health Survey Report. Sydney, NSW: Justice Health and Forensic Mental Health Network (2017).

[24] Australian Bureau of Statistics. Estimates and projections, Aboriginal and Torres Strait Islander Australians, 2006 to 2031. Cat. no. 3238.0. Canberra: ABS (2019).

[25] Hancock N Smith-Merry J Mckenzie K. Facilitating people living with severe and persistent mental illness to transition from prison to community: a qualitative exploration of staff experiences. Int J Ment Health Syst. (2018) 12:1–10. 10.1186/s13033-018-0225-z 30116292 PMC6085690

[26] Butler A Love AD Young JT Kinner SA. Frequent attendance to the emergency department after release from prison: a prospective data linkage study. J Behav Health Serv Res. (2020) 47:544–59. 10.1007/s11414-019-09685-1 31820327 PMC7578130

[27] Alan J Burmas M Preen D Pfaff J. Inpatient hospital use in the first year after release from prison: a Western Australian population-based record linkage study. Aust N Z J Public Health. (2011) 35:264–9. 10.1111/j.1753-6405.2011.00704.x 21627727

[28] Dean K Browne C Dean N. Stigma discrimination experiences amongst those with mental illness in contact with the Criminal Justice System. Rapid review report for the National Mental Health Commission. (2022). Available online at: https://haveyoursay.mentalhealthcommission.gov.au/72951/widgets/363554/documents/224742 (accessed August 12, 2022).

[29] Morris NP. “Use the back door”: treating incarcerated patients in community mental health facilities. Psychiatr Serv. (2019) 70:967–70. 10.1176/appi.ps.201900344 31434560

[30] Fovet T Lamer A Teston R Scouflaire T Thomas P Horn M. Access to a scheduled psychiatric community consultation for prisoners with mood disorders during the immediate post-release period. J Affect Disord Rep. (2021) 4:100088. 10.1016/j.jadr.2021.100088

[31] Dean K Korobanova D. Brief mental health screening of prison entrants: psychiatric history versus symptom screening for the prediction of in-prison outcomes. J Forens Psychiatry Psychol. (2018) 29:455–66. 10.1080/14789949.2017.1421247

[32] Walker J Illingworth C Canning A Garner E Woolley J Taylor P. Changes in mental state associated with prison environments: a systematic review. Acta Psychiatr Scand. (2014) 129:427–36. 10.1111/acps.12221 24237622

[33] Baillargeon J Penn JV Knight K Harzke AJ Baillargeon G Becker EA. Risk of reincarceration among prisoners with co-occurring severe mental illness and substance use disorders. Adm Policy Ment Health. (2010) 37:367–74. 10.1007/s10488-009-0252-9 19847638

[34] Australian Productivity Commission. Steering Committee for the Review of Government Service Provision. Report on Government Services 2021. (2021). Availble online at: https://www.pc.gov.au/research/ongoing/report-on-government-services/2021 (accessed February 17, 2022).

[35] Browne CC Korobanova D Yee N Spencer SJ Ma T Butler T. The prevalence of self-reported mental illness among those imprisoned in New South Wales across three health surveys, from 2001 to 2015. Aust N Z J Psychiatry. (2022). 10.1177/00048674221104411 [Epub ahead of print]. 35694738

