# Supplementary material for: Continuity of mental health care during the transition from prison to the community following brief periods of imprisonment
Source: Front Psychiatry. 2022 Sep 20;13:934837. doi: 10.3389/fpsyt.2022.934837 (PMC9530150; doi:10.3389/fpsyt.2022.934837)
Supplement: Supplementary file 1 [file Data_Sheet_1.pdf]

Supplementary File 1: Prison Mental Health Screening (PMHS) Tool

MENTAL HEALTH & SELF-HARM/SUICIDE RECEPTION SCREENING– STAGE I TOOL

MENTAL HEALTH HISTORY

1. Prior to coming into prison have you ever had ...
(*Prompt with the following possible diagnoses)*

		Yes	In the last month?	No	Unsure/Don't know	Declined to Answer
a.	Depression	☐1	☐2	☐0	☐3	☐
b.	Anxiety	☐1	☐2	☐0	☐3	☐
c.	Schizophrenia	☐1	☐2	☐0	☐3	☐
d.	Bipolar disorder (manic depression)	☐1	☐2	☐0	☐3	☐
e.	Drug-induced psychosis	☐1	☐2	☐0	☐3	☐
f.	Other mental health problems?	☐1	☐2	please specify:		

2. Have you ever received treatment for a mental health problem including those you've identified in the previous question?

Yes ☐1	**What type of treatment did you receive?**	Medication ☐<br>Talking therapy (e.g., counselling) ☐<br>Seen by a psychiatrist ☐<br>Other ☐<br>Don't know ☐<br>Declined to answer ☐
No ☐0<br>Unsure/Don't Know ☐2<br>Declined to answer ☐		

3. Have you ever been admitted to hospital for a mental health problem?

Yes ☐1 No ☐0 Unsure/Don't Know ☐2 Declined to answer ☐

FAMILY HISTORY

4. Has anyone in your family ever suffered from a mental health problem?

Yes ☐1 No ☐0 Unsure/Don't Know ☐2 Declined to answer ☐

Specify who/what:

Participant Study ID

☐☐☐☐

MENTAL HEALTH SYMPTOMS

**Have you ever...** (*prompt with possible symptoms)*	Yes	In the last month?	No	Unsure/ Don't know	Declined to Answer
a. Heard things that other people couldn't, such as noises, or the voices of people whispering or talking; OR had visions or saw things that other people couldn't see?	☐1	☐2	☐0	☐3	☐
b. Thought or felt that someone is going out of their way to give you a hard time, or trying to hurt you?	☐1	☐2	☐0	☐3	☐
c. Felt that you were especially important in some way, or had special powers to do things that other could not do?	☐1	☐2	☐0	☐3	☐
d. Felt as if your thoughts were being broadcast out loud so that other people could actually hear what you were thinking; or believed that someone could read your mind?	☐1	☐2	☐0	☐3	☐
e. Felt that you're not in control of your own ideas or thoughts or felt as though another person or force was interfering with your thoughts?	☐1	☐2	☐0	☐3	☐
f. Seen special meanings in advertisements, shop windows, in the way things are arranged around you or received messages from the TV or the radio?	☐1	☐2	☐0	☐3	☐
g. Felt depressed or down most of the day, nearly every day for at least two weeks?	☐1	☐2	☐0	☐3	☐
h. Lost interest or pleasure in things you usually enjoyed, nearly every day for at least two weeks?	☐1	☐2	☐0	☐3	☐
i. Experienced a period of time when you were feeling so good, "high", excited, or hyper that other people thought you were not your normal self?	☐1	☐2	☐0	☐3	☐
j. Found it hard to do your usual work, take care of things at home, or get along with other people for a sustained period?	☐1	☐2	☐0	☐3	☐
k. Had difficulty thinking or concentrating, or making decisions about everyday things?	☐1	☐2	☐0	☐3	☐
l. Experienced a change in sleep (trouble falling asleep, waking frequently, trouble staying asleep, waking too early, sleeping too much, needing less sleep than usual and still feeling rested)	☐1	☐2	☐0	☐3	☐

Participant Study ID

☐☐☐☐

SUICIDE AND SELF-HARM RISK ASSESSMENT

1. Have you ever tried to hurt yourself?

☐1 Yes → **a, b**

☐0 No

☐2 Unsure

☐ Declined to answer

a. When was the most recent occasion?

☐1 In the past week
☐2 1 week -< 4 weeks ago
☐3 1 month -< 6 months ago
☐4 6 months -< 1 year ago
☐5 More than 1 year ago

b. What did you do?

☐ Poisoning/Overdose
☐ Laceration/Cutting/Slashing
☐ Burning
☐ Hanging/strangulation
☐ Hitting self/jumping from heights
☐ Swallowing objects
☐ Pulled/picked on wounds
☐ Not eating/drinking
☐ Other (specify)

---

2. Have you ever tried to end your life?

☐1 Yes → **a, b**

☐0 No

☐2 Unsure

☐ Declined to answer

a. When was the most recent occasion?

☐1 In the past week
☐2 1 week -< 4 weeks ago
☐3 1 month -< 6 months ago
☐4 6 months -< 1 year ago
☐5 More than 1 year ago

b. What did you do?

☐ Hanging
☐ Overdose/poisoning
☐ Firearms/gunshot
☐ Slashing/stabbing
☐ Drowning
☐ Jumping from heights
☐ Other (specify)

**3. In the past week, have you**	Yes	No	Unsure/ Don't know	Declined to Answer
a. Been thinking that you might be better off dead?	☐1	☐0	☐2	☐
b. Had any thoughts that life is not worth living?	☐1	☐0	☐2	☐
c. Had thoughts about hurting or killing yourself?	☐1	☐0	☐2	☐
d. Had thoughts about how you might end your life?	☐1	☐0	☐2	☐

4. Are there significant stressors that cause you concern outside of prison?

No ☐0 Unsure ☐2 Declined to answer ☐

Yes ☐1 Relationship difficulties/Family Difficulties/Disability or Illness/Assault/Loss or Grief/Children/Court or Legal/ Other (specify)

---

5. Is this your first time in prison?

Yes ☐1 No ☐0 Declined to answer ☐

---

6. Do you think you will be able to cope in prison?

Yes ☐1 No ☐0 Unsure ☐2 Declined to answer ☐

---

7. Interviewer's observation of patient, including whether the Mental Health Screening outcome is consistent with the patient's presentation (behaviour, mood, responses, refer to Reception Triage Charts for outline of mental state examination). (e.g., poor eye contact, disorganised speech/behaviour, not able to stay on topic, mute or impossible to interrupt)

Does the person appear to be intoxicated or are you aware that they are intoxicated? Yes ☐1 No ☐0

Have you observed something in the behaviour that makes you believe the person requires a Stage II Mental Health Assessment? Yes ☐ No ☐

If **Yes**, please describe the behaviour:
