# Supplementary material for: Continuity of mental health care during the transition from prison to the community following brief periods of imprisonment
Source: Front Psychiatry. 2022 Sep 20;13:934837. doi: 10.3389/fpsyt.2022.934837 (PMC9530150; doi:10.3389/fpsyt.2022.934837)
Supplement: Supplementary file 2 [file Data_Sheet_2.pdf]

| File Check | | | | | |
|---|---|---|---|---|---|
| Aboriginal/Torres Strait Islander status | 1= Aboriginal | 2= Torres Strait Islander | | 3=Neither | 4 = Both |
| Legal status | 1= Remand | 2= Sentenced | | 3= Forensic patient | |
| Date of current incarceration | / / | | | | |
| First time in custody? | 0= No | 1= Yes | | | |
| Current charges? | | | | | |
| I/O type | 1= Non-Viol | 2= Violent | | | |
| Was a victim involved? | 0= No | 1= Yes | | | |
| If yes, who… | 1= Acquaintance | 2= Family | 3= Stranger | | |
| Was a weapon involved? | 0= No | 1= Yes | | | |
| If yes, what… | 1= Knife | 2= Gun | 3= Other | | |
| Age at first time in custody: | | years | | | |
| Date of alleged index offence? | / / | | | | |
| Diagnoses (as recorded in file; include all): | Dx 1 = | | Dx 6 = | | |
| (*including substance abuse disorders)* | Dx 2 = | | Dx 7 = | | |
| | Dx 3 = | | Dx 8 = | | |
| | Dx 4 = | | Dx 9 = | | |
| | Dx 5 = | | Dx 10 = | | |
| Next court date | / / | | | | |
| Court location | | | | | |
| Previous State-wide Court & Community Liaison contact (if any) | | | | | |
| Previous Community Mental Health details: | | | | | |
| Keyworker Name: | | | | | |
| CMHT Name: | | | | | |
| Keyworker contact details: | Email:<br>Phone:<br>Mobile: | | | | |
| GP Name: | | | | | |
| GP Contact details: | Email:<br>Phone:<br>Mobile: | | | | |
| Parole/Probation Details: | | | | | |
| Earliest release date: | / / | | | | |
| Release location: | | | | | |
| Release conditions: | | | | | |
