# Supplementary material for: Continuity of mental health care during the transition from prison to the community following brief periods of imprisonment
Source: Front Psychiatry. 2022 Sep 20;13:934837. doi: 10.3389/fpsyt.2022.934837 (PMC9530150; doi:10.3389/fpsyt.2022.934837)
Supplement: Supplementary file 3 [file Data_Sheet_3.pdf]

Participant Follow-Up Interview

**Date of the interview:**	____/____/____
**Method of contact:**	1 = Phone<br>2 = Face to face

Administer the following items from Stage I Tool Mental Health Section Question 5:

Have you ever… (*prompt with possible symptoms)*

	Yes	In the last month?	No	Unsure/ Don't know	Declined to Answer
1. Heard things that other people couldn't, such as noises, or the voices of people whispering or talking; OR had visions or saw things that other people couldn't see?	☐1	☐2	☐0	☐3	☐9
2. Thought or felt that someone is going out of their way to give you a hard time, or trying to hurt you?	☐1	☐2	☐0	☐3	☐9
3. Felt that you were especially important in some way, or had special powers to do things that other could not do?	☐1	☐2	☐0	☐3	☐9
4. Felt as if your thoughts were being broadcast out loud so that other people could actually hear what you were thinking; or believed that someone could read your mind?	☐1	☐2	☐0	☐3	☐9
5. Felt that you're not in control of your own ideas or thoughts or felt as though another person or force was interfering with your thoughts?	☐1	☐2	☐0	☐3	☐9
6. Seen special meanings in advertisements, shop windows, in the way things are arranged around you or received messages from the TV or the radio?	☐1	☐2	☐0	☐3	☐9
7. Felt depressed or down most of the day, nearly every day for at least two weeks?	☐1	☐2	☐0	☐3	☐9
8. Lost interest or pleasure in things you usually enjoyed, nearly every day for at least two weeks?	☐1	☐2	☐0	☐3	☐9
9. Experienced a period of time when you were feeling so good, "high", excited, or hyper that other people thought you were not your normal self?	☐1	☐2	☐0	☐3	☐9
10. Found it hard to do your usual work, take care of things at home, or get along with other people for a sustained period?	☐1	☐2	☐0	☐3	☐9
11. Had difficulty thinking or concentrating, or making decisions about everyday things?	☐1	☐2	☐0	☐3	☐9
12. Experienced a change in sleep (trouble falling asleep, waking frequently, trouble staying asleep, waking too early, sleeping too much, needing less sleep than usual and still feeling rested)?	☐1	☐2	☐0	☐3	☐9

**Contact with CMHT: In the last 3 months…**	
Have you been in contact with CMHT?	0 = No<br>1 = Yes
If YES:	
Date of first contact:	____/____/____
Number of times in contact:	
If more than one contact, date of last:	____/____/____
Types of contact:	1 = Phone<br>2 = Face to face<br>3 = Home visit<br>4 = Other (specify): ________________________________
Who have you seen?	1 = Keyworker<br>2 = Social worker<br>3 = Occupational therapist<br>4 = Psychiatrist<br>5 = Other (specify): ________________________________
If NO:	
What were the reasons for not being in contact with CMHT keyworker?	

**Mental Health Care and Unmet Need**		
In the last 3 months, did you receive any information about mental illness, treatment, and services available?	0 = No<br>1 = Yes	8 = DK<br>9 = Declined
**If YES**, did the information provided to you meet your needs?	0 = No<br>1 = Yes	8 = DK<br>9 = Declined
**If NO**, would you have liked to have received information about mental illness, treatment, and available services?	0 = Do not want this information<br>1 = Would have liked to have this information<br>9 = Declined	
In the last 3 months, did you receive any therapy where you and your keyworker or other clinician explored your thoughts, feelings, and beliefs about your symptoms and illness and came up with new ways of understand them and coping?	0 = No<br>1 = Yes	8 = DK<br>9 = Declined
**If YES**, did this counselling or talking therapy provided to you meet your needs?	0 = No<br>1 = Yes	8 = DK<br>9 = Declined
**If NO**, would you have liked to have received this counselling or talking therapy?	0 = No<br>1 = Yes	8 = DK<br>9 = Declined
In the last 3 months, have you and your family met together regularly with a mental health clinician to learn about mental illness and improve your communication and problem-solving skills?	0 = No<br>1 = Yes	8 = DK<br>9 = Declined
**If YES**, how helpful did you find these meetings?	1 = Very helpful<br>2 = Somewhat helpful<br>3 = Neither helpful nor unhelpful<br>4 = Somewhat unhelpful<br>5 = Very unhelpful<br>8 = DK<br>9 = Declined	
In general, in the last 3 months did you experience a need for a particular kind of service or helpful in any way but were unable to get it, perhaps because it was unavailable?	0 = No<br>1 = Yes	8 = DK<br>9 = Declined
How would you rate your **overall** mental health?	1 = Excellent	8 = DK

	2 = Very good 9 = Declined<br>3 = Good<br>4 = Fair<br>5 = Poor
**Substance Use**	
Have you ever had an alcoholic drink?	0 = No 8 = DK<br>1 = Yes 9 = Declined
**If YES**, in the last 3 months:	
How often did you have an alcoholic drink?	0 = Never<br>1 = Monthly or less<br>2 = 2 to 4 times a month<br>3 = 2 to 3 times a week<br>4 = 4 or more times a week<br>8 = DK<br>9 = Declined
How many standard drinks did you usually have on a typical day when you were drinking?	0 = 1 or 2<br>1 = 3 or 4<br>2 = 5 or 6<br>3 = 7 to 9<br>4 = 10 or more<br>8 = DK<br>9 = Declined
How often did you have six or more drinks on one occasion?	0 = Never<br>1 = Less than monthly<br>2 = Monthly<br>3 = Weekly<br>4 = Daily/almost daily<br>8 = DK<br>9 = Declined
Have you ever used illegal drugs or non-prescribed medication repeatedly?	0 = No 8 = DK<br>1 = Yes 9 = Declined
**If YES**, in the last 3 months:	
What drugs have you used? *(allow multiple responses)*	1 = Misuse of painkillers/prescribed analgesics, tranquilizers/sleeping pills, methadone/Buprenorphine<br>2 = Heroin<br>3 = Cannabis/marijuana<br>4 = Methamphetamine (ice), amphetamine (speed)<br>6 = Cocaine<br>7 = Ecstasy<br>8 = GHB<br>9 = LSD, hallucinogens<br>10 = Other drugs (synthetic drugs, steroids, amyl nitrate, petrol/solvents, ketamine, barbiturates)<br>88 = DK<br>99 = Declined
Did you use any of these drugs daily?	0 = No 8 = DK<br>1 = Yes 9 = Declined
Were you intoxicated at time of the alleged offence?	0 = No 8 = DK<br>1 = Yes 9 = Declined
If yes, what substance(s) were you under the influence of at the time of the alleged offence? *(allow multiple responses)*	
**Medication Use**	
Are you currently taking any	0 = No 8 = DK

medication for your psychiatric/mental health?	1 = Yes	9 = Declined
**If YES,**		
Do you feel your medication eases your mental health symptoms?	1 = A lot<br>2 = A little<br>3 = Not at all	8 = DK<br>9 = Declined
What do you think would happen if you stopped taking your medication?	0 = Nothing would happen<br>1 = I would become unwell<br>2 = Other (specify)__________________	8 = DK<br>9 = Declined

**Daily Functioning** *(adapted from WHODAS 2.0)*					
In the last 30 days, how much difficulty did you have in:	None	Mild	Moderate	Severe	Extreme or can't do
Showering, getting dressed, or eating?	0	1	2	3	4
Taking care of your household responsibilities?	0	1	2	3	4
Remembering to do important things?	0	1	2	3	4
Maintaining close relationships with family and friends?	0	1	2	3	4
Managing my finances?	0	1	2	3	4
Are you currently working or studying?	0 = No<br>1 = Yes	8 = DK<br>9 = Declined			

**Experiences since release**	
Have you engaged in any criminal activity, including violence, since your release?	0 = No<br>1 = Yes
Specify:	
Have you been a victim of crime, abuse, trauma or other type of victimisation since your release?	0 = No<br>1 = Yes
Specify:	
